# Emerging roles for dynamic aquaporin-4 subcellular relocalization in CNS water homeostasis

**DOI:** 10.1093/brain/awab311

**Published:** 2021-09-09

**Authors:** Mootaz M Salman, Philip Kitchen, Andrea Halsey, Marie Xun Wang, Susanna Törnroth-Horsefield, Alex C Conner, Jerome Badaut, Jeffrey J Iliff, Roslyn M Bill

**Affiliations:** 1 Department of Physiology, Anatomy and Genetics, University of Oxford, Oxford OX1 3PT, UK; 2 School of Biosciences, College of Health and Life Sciences, Aston University, Aston Triangle, Birmingham B4 7ET, UK; 3 Institute of Clinical Sciences, College of Medical and Dental Sciences, University of Birmingham, Edgbaston, Birmingham B15 2TT, UK; 4 Department of Psychiatry and Behavioral Sciences, University of Washington School of Medicine, Seattle, WA, USA; 5 Department of Biochemistry and Structural Biology, Lund University, PO Box 124, 221 00 Lund, Sweden; 6 CNRS-UMR 5536-Centre de Résonance Magnétique des systèmes Biologiques, Université de Bordeaux, 33076 Bordeaux, France; 7 Department of Neurology, University of Washington School of Medicine, Seattle, WA, USA; 8 VISN 20 Mental Illness Research, Education and Clinical Center, VA Puget Sound Health Care System, Seattle, WA, USA

**Keywords:** water channel, regulation, traumatic brain and spinal cord injury, neurodegeneration

## Abstract

Aquaporin channels facilitate bidirectional water flow in all cells and tissues. AQP4 is highly expressed in astrocytes. In the CNS, it is enriched in astrocyte endfeet, at synapses, and at the glia limitans, where it mediates water exchange across the blood–spinal cord and blood–brain barriers (BSCB/BBB), and controls cell volume, extracellular space volume, and astrocyte migration. Perivascular enrichment of AQP4 at the BSCB/BBB suggests a role in glymphatic function. Recently, we have demonstrated that AQP4 localization is also dynamically regulated at the subcellular level, affecting membrane water permeability. Ageing, cerebrovascular disease, traumatic CNS injury, and sleep disruption are established and emerging risk factors in developing neurodegeneration, and in animal models of each, impairment of glymphatic function is associated with changes in perivascular AQP4 localization. CNS oedema is caused by passive water influx through AQP4 in response to osmotic imbalances. We have demonstrated that reducing dynamic relocalization of AQP4 to the BSCB/BBB reduces CNS oedema and accelerates functional recovery in rodent models. Given the difficulties in developing pore-blocking AQP4 inhibitors, targeting AQP4 subcellular localization opens up new treatment avenues for CNS oedema, neurovascular and neurodegenerative diseases, and provides a framework to address fundamental questions about water homeostasis in health and disease.

## Introduction

**Figure 1:**
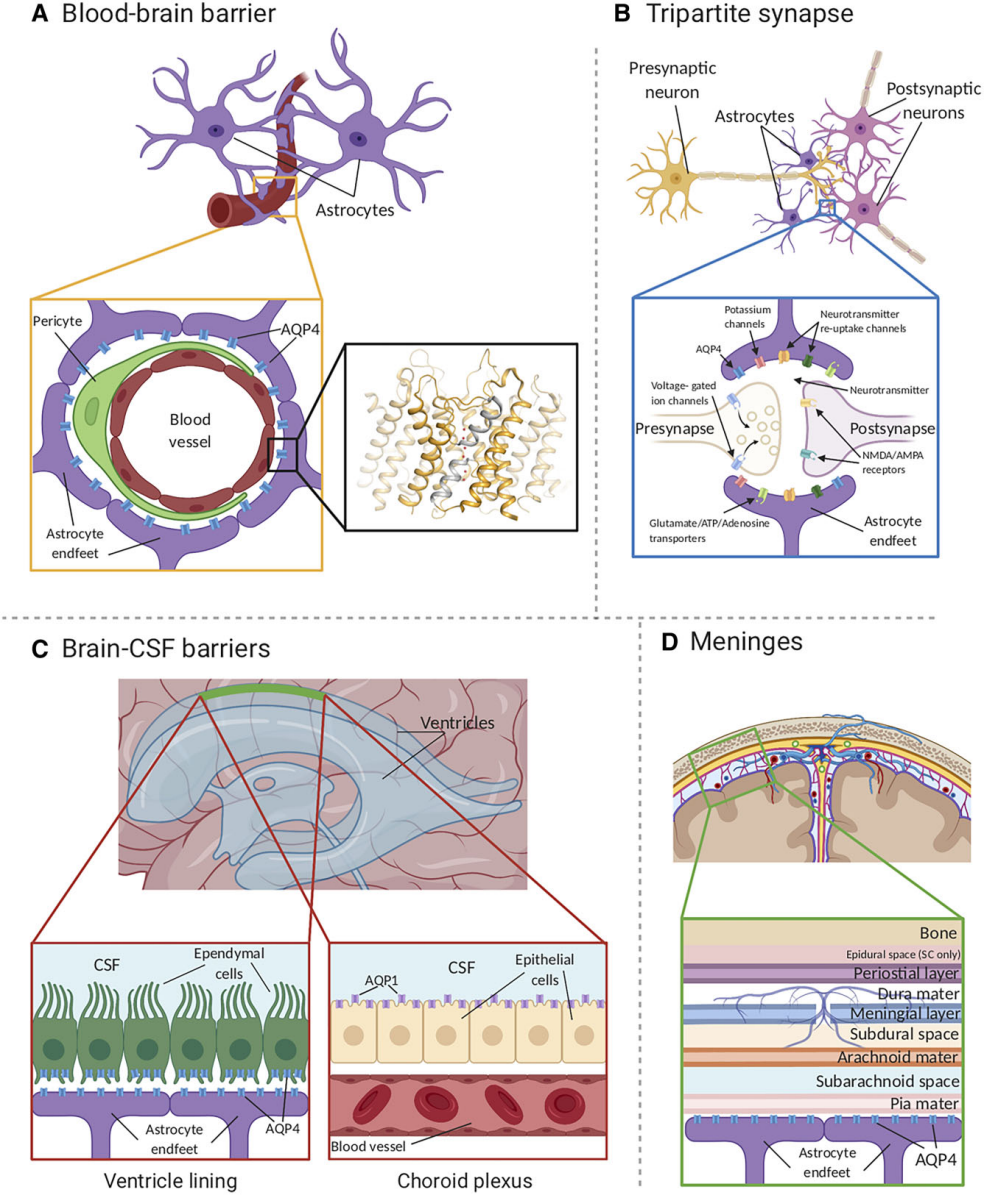
**AQP4 localization in the CNS.** (**A**) AQP4 (blue) is located within astrocyte endfeet processes surrounding blood vessels in both brain tissue and the BBB. The *inset* shows the crystal structure of human AQP4 (PDB code 3GD8). AQP4 assembles as a tetramer with each monomer comprising six transmembrane helices and two half-helices (grey). The two half helices harbour the aquaporin signature motif (NPA) as well as part of the aromatic-arginine (ar/R) motif that functions as a selectivity filter. Within the pore, water molecules (red spheres) align in a single file. (**B**) AQP4 is localized at the astrocyte component of the tripartite synapse. During neurotransmission, neurons release mediators and neurotransmitters from synaptic nerve terminals (affecter cells) into the synaptic cleft to communicate with other neurons (effector cells). This synaptic activity induces an increase in intracellular Ca^2+^ concentration, which is accompanied by changed water and solute concentrations in astrocytes, leading to the release of glutamate and other gliotransmitters. This gliotransmission results in negative feedback to the presynaptic neurons to modulate neurotransmission. AQP4 plays an essential role in maintaining water homeostasis during this process. (**C**) In ventricles, AQPs are present within ependymal cells lining the brain-CSF interfaces (*left inset*). AQP4 is localized to the basolateral membrane of ependymal cells and the endfeet of contacting astrocytes (*right inset*). AQP1 (purple) is localized to the apical membrane of the choroid plexus epithelium.^6^^,^^30^ (**D**) CSF within the subarachnoid and cisternal spaces flows into the brain specifically via periarterial spaces and then exchanges with brain interstitial fluid facilitated by AQP4 water channels that are positioned within perivascular astrocyte endfoot processes.

The control of water homeostasis is crucial in maintaining normal CNS function. Dysregulation results in rapid and potentially life-threatening increases in intracranial or intraspinal pressure,^1^^,^^2^ or the accumulation of toxic waste products.^3^ Of the three aquaporins described in the CNS (AQP1, 4 and 9), AQP4 is the most abundant. It is found in astrocytes and is enriched at the blood–spinal cord and blood–brain barriers (BSCB/BBB), tripartite synapses, ventricle lining and the glia limitans beneath the meninges (Fig. 1). Studies in transgenic mice have established that AQP4 is a major regulator of CNS water homeostasis,^4^^,^^5^ where it controls the exchange of CSF with brain interstitial fluid and facilitates the development (and may also facilitate the clearance) of CNS oedema.^6^

Aquaporin channels facilitate the bidirectional flow of water and small uncharged solutes, whose membrane permeability is controlled by aquaporin abundance.^7^^,^^8^ The structural biology of aquaporin transmembrane domains is well-established^9^: six membrane-spanning α-helices and two half-helices stack around the family’s signature Asn-Pro-Ala (NPA) motifs (located in the middle of the membrane) to form the water pore (Fig. 1A, inset). Members of the aquaporin family can be selective for water (e.g. AQP4) or also permit the transport of small neutral solutes such as glycerol and urea (e.g. AQP9).^10^ The substrate traverses the pore in single file, charged species are excluded by the channel electrostatics, and protons are excluded by the orientation of water molecules within the pore preventing proton diffusion along the hydrogen bond network via the Grotthuss mechanism. Less is known about the structures of the intracellular amino- and carboxy-termini, which are not usually resolved in crystallography studies,^9^ but where many key regulatory interactions are known to occur. Aquaporins are homotetramers, with each monomer containing an independent water pore. The functional relevance of the tetramer is unclear, although we have shown that AQP4 mutants that do not tetramerize are also unable to relocalize to the plasma membrane.^11^

AQP4 exists in two major isoforms, namely AQP4-M1 and AQP4-M23 (indicating the position of the initiating methionine residue). The shorter AQP4-M23 isoform can be derived from an alternatively-spliced transcript,^12^ or by leaky-scanning of the M1 transcript whereby the 40S ribosome skips the first (M1) start codon and initiates translation at the second (M23).^13^ AQP4-M23 forms square arrays in the astrocyte plasma membrane, known as orthogonal arrays of particles (OAPs).^14^ These OAPs can be observed directly by freeze fracture electron microscopy.^15^ OAP size depends upon the ratio between AQP4-M1 and AQP4-M23, with higher levels of AQP4-M1 composition reducing OAP size. Notably, OAP disintegration and changes in the ratio between AQP4-M1 and AQP4-M23 are observed early after stroke,^16-18^ although the (patho)physiological consequences of these changes are yet to be defined. Recent work suggests that OAP stability can impact astrocyte process motility and local synaptic activity.^19^ A better understanding of OAPs may be possible in the future with the development of a novel mouse lacking the OAP-forming AQP4-M23 isoform.^20^^,^^21^ An AQP4 isoform (AQPex) has also been reported that has an extended carboxy-terminus containing a conserved perivascular localization signal generated by translational read-through.^22^^,^^23^ The consequences of this carboxy-terminal extension are yet to be established.

The notable localization of AQP4 to perivascular astrocyte endfoot processes results from its association with the dystrophin-associated complex (DAC), which anchors AQP4 intracellularly to the cytoskeleton and extracellularly to the cerebrovascular basal lamina. Deletion of the *Dmd* and *Snta1* genes (which encode the DAC proteins, dystrophin and α-syntrophin), or of *Agrn* (which encodes the basal lamina protein, agrin) in mice results in the loss of this perivascular AQP4 localization.^24–27^ A recent study also suggests a potential role for β-syntrophin in AQP4 anchoring.^28^ Changes in perivascular localization of AQP4 have been reported across myriad pathological conditions, including CNS tumours, neurovascular disorders, such as ischaemic stroke and traumatic brain injury, and in the setting of neurodegenerative disease.^29^ Perivascular localization of AQP4 may also be regulated by differential regulation of AQP4-M1 versus AQP4-M23 expression, with AQP4-M23-enriched OAPs localizing to perivascular astroglial endfoot processes.^20^^,^^31^ The degree of enrichment of AQP4 to perivascular membranes differs between brain regions, although the molecular basis and physiological consequences of these differences remains incompletely understood.^32^

While studies of AQP4 function have historically focused on this cell-level localization to perivascular processes, more recent work from our group suggests that dynamic subcellular relocalization of AQP4, from intracellular vesicles to the plasma membrane, may play a crucial role in the regulation of AQP4 function.^7^ The plasma membrane abundance of most mammalian aquaporins has been shown to respond to distinct cellular or environmental triggers, such as hormones or changes in tonicity.^33^ This is best described for AQP2, for which trafficking in response to the pituitary hormone arginine vasopressin (AVP) involves regulated exocytosis of AQP2-containing storage vesicles in the kidney collecting duct principal cells.^34^ Although the specific triggers will vary between isoforms and cell types, studies indicate that the dynamic subcellular relocalization of human aquaporins share several features: (i) a trigger causing a signalling cascade leading to site-specific aquaporin phosphorylation; (ii) the subsequent movement of aquaporin-containing vesicles along the microtubule network; and (iii) vesicle fusion with the plasma membrane.^35^ Our recent work has shown that AQP4 plasma membrane abundance is tightly and dynamically regulated at the subcellular level by relocalization to and from intracellular vesicular pools in response to non-hormonal stimuli in astrocyte cultures.^7^^,^^36^^,^^37^ These include the changes in local oxygen tension and osmolality that are caused by traumatic injury and stroke. Targeting this regulatory mechanism is a viable anti-oedema therapy in rodent models of spinal cord injury, traumatic brain injury and stroke.^7^^,^^38^

Pore-blocking molecules for aquaporins remain difficult to develop. The small diameter of the aquaporin pore (water molecules traverse the pore in single file), the fact that interactions are limited to hydrogen bonding^39^ and a lack of *in vitro* assays suitable for screening and validating the pharmacological regulation of aquaporin function^40^ are all factors. This lack of tool compounds to modulate aquaporin function means that many fundamental questions about water homeostasis remain unanswered. Here we review the physiological and pathophysiological roles of AQP4 in the CNS, with a focus on novel insights into the mechanisms of glymphatic clearance in the maintenance of brain water homeostasis and new approaches to drug discovery that can be derived from the discovery of dynamic AQP4 subcellular relocalization.

## Physiological roles of AQP4: the glymphatic pathway

**Figure 2:**
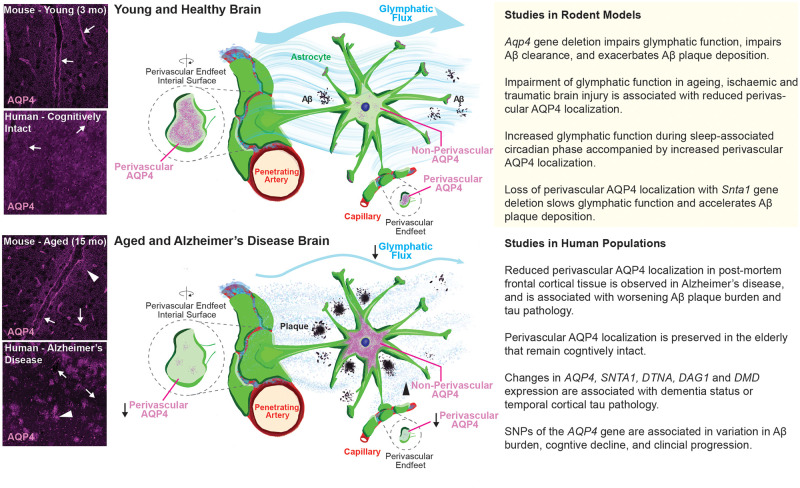
**The glymphatic pathway.** The glymphatic system is a perivascular network that facilitates fluid exchange between the CSF and interstitial compartments, supporting the clearance of interstitial solutes. The function of the glymphatic system relies on perivascular astrocyte AQP4 expression. In the healthy young brain, AQP4 localizes to the astrocyte endfeet along the perivascular space (*top left*, arrows). In the context of ageing and Alzheimer’s disease, perivascular AQP4 levels are reduced while cellular AQP4 levels are increased (*bottom left*, arrows). The loss of AQP4 from perivascular astrocytic endfeet slows glymphatic clearance, which may accelerate amyloid-β accumulation and cognitive decline. The column on the *right* details specific findings from studies in rodents (*top*) and humans (*bottom*).

Since 2012,^41^ AQP4 has been implicated as a key determinant of glymphatic function (Fig. 2). The glymphatic system (recently and comprehensively reviewed by Rasmussen and colleagues^42^) is a brain-wide network of perivascular pathways along which CSF enters the brain and interstitial solutes are cleared.^43^^,^^44^ Glymphatic exchange is driven by arterial pulsation,^45^^,^^46^ is active primarily during sleep,^47–49^ and contributes to the clearance of interstitial amyloid-β,^41^^,^^49^ tau^50^^,^^51^ and other solutes such as lactate,^52^ and inflammatory cytokines.^53^

Both glymphatic influx of CSF and interstitial solute clearance are dependent upon perivascular AQP4. In the initial description of the glymphatic system,^41^ *Aqp4* gene deletion was observed to slow CSF tracer influx and interstitial tracer efflux in mice. Similarly, deletion of the *Aqp4* gene slowed the clearance of amyloid-β from the brain,^41^ and promoted the formation of amyloid plaques.^54^ Although one study failed to reproduce this effect of *Aqp4* gene deletion on CSF tracer distribution,^55^ a subsequent study reporting data from five independent laboratories using five different transgenic mouse lines confirmed the role of AQP4 in perivascular glymphatic exchange.^56^ In that study, *Stna1* gene deletion was observed to impair glymphatic function, demonstrating that perivascular localization of AQP4 plays a critical role in AQP4-dependent glymphatic exchange.

Under physiological conditions in mice, increasing perivascular AQP4 levels during rest and declining perivascular AQP4 levels during activity were associated with increased, and reduced glymphatic function, respectively.^47^ Pathologically, glymphatic function is impaired in ageing mice,^57^ following traumatic brain injury,^50^ and in rodent models of cerebrovascular disease.^58–60^ In each case, impairment of perivascular exchange was associated with a reduction in the cell-level localization of AQP4 to perivascular processes.^57^^,^^61^^,^^62^ When this perivascular localization of AQP4 is disrupted by deletion of the *Snta1* gene, glymphatic function is similarly impaired.^56^ While these findings suggest that one of the roles of perivascular AQP4 is to facilitate the exchange of CSF and interstitial fluid along the axis of the cerebral vasculature, thereby supporting solute distribution and waste clearance, the mechanism controlling changes in the cell-level localization of AQP4 to perivascular endfeet under physiological and pathological conditions remains to be established. Importantly, these studies defining the role of perivascular AQP4 in glymphatic function have not clearly distinguished between AQP4 pools inserted into the endfoot plasma membrane and those in sub-membrane vesicles. The manner in which cell-level changes in perivascular AQP4 localization interact with the recently described dynamic subcellular changes in AQP4 abundance^7^ to govern glymphatic function remains to be explored.

## Pathological roles of AQP4

### Neurodegenerative disease

Ageing, cerebrovascular disease, prior exposure to traumatic brain injury, and sleep disruption are established and emerging risk factors for the development of neurodegenerative conditions, including Alzheimer’s disease. In animal models of each, glymphatic function is impaired.^50^^,^^57–60^^,^^63^ Given the role of perivascular glymphatic exchange in amyloid-β^41^^,^^49^ and tau^50^^,^^51^ clearance, impairment of glymphatic pathway function is now proposed to be important in the development of these conditions.^64^ While imaging of glymphatic function using dynamic contrast-enhanced MRI (DCE-MRI) has only recently begun,^43^^,^^65^ early studies demonstrate that glymphatic function in humans is impaired in normal-pressure hydrocephalus^44^^,^^66^ and in the presence of small vessel disease.^67^

The role of glymphatic impairment in the development of other neurodegenerative diseases has not yet been directly evaluated, but emerging data from studies in human populations suggest a role for AQP4 in these conditions. In a post-mortem case series,^68^ reduced perivascular AQP4 abundance was observed in the frontal cortex of subjects diagnosed with Alzheimer’s disease, while preservation of perivascular AQP4 abundance was observed in subjects remaining cognitively intact over the age of 85. The reduced perivascular AQP4 abundance was further associated with increasing amyloid-β and tau pathology, as well as with global measures of cognitive decline. In three recent genetics studies carried out in distinct human populations, single nucleotide polymorphisms in the human *AQP4* gene were associated with variation in cognitive decline,^69^ amyloid burden and clinical status,^70^ and an association between sleep disruption and amyloid burden.^71^ A recent human transcriptomic study further demonstrated that in addition to the expression of *AQP4*, differences in the expression of genes whose products determine perivascular AQP4 localization (specifically genes encoding elements of the DAC, *SNTA1*, *DTNA*, *DMD*, *DAG1*) were associated with dementia status and temporal cortical tau pathology.^72^ These findings suggest that changes in AQP4 expression and localization may contribute to the development and progression of neurodegenerative diseases, including Alzheimer’s disease, in human populations. Understanding the emerging role of dynamic AQP4 subcellular relocalization provides a new framework to understand waste clearance in the healthy brain and opens up new treatment avenues to slow the progression of neurodegenerative diseases.

### CNS oedema

**Figure 3:**
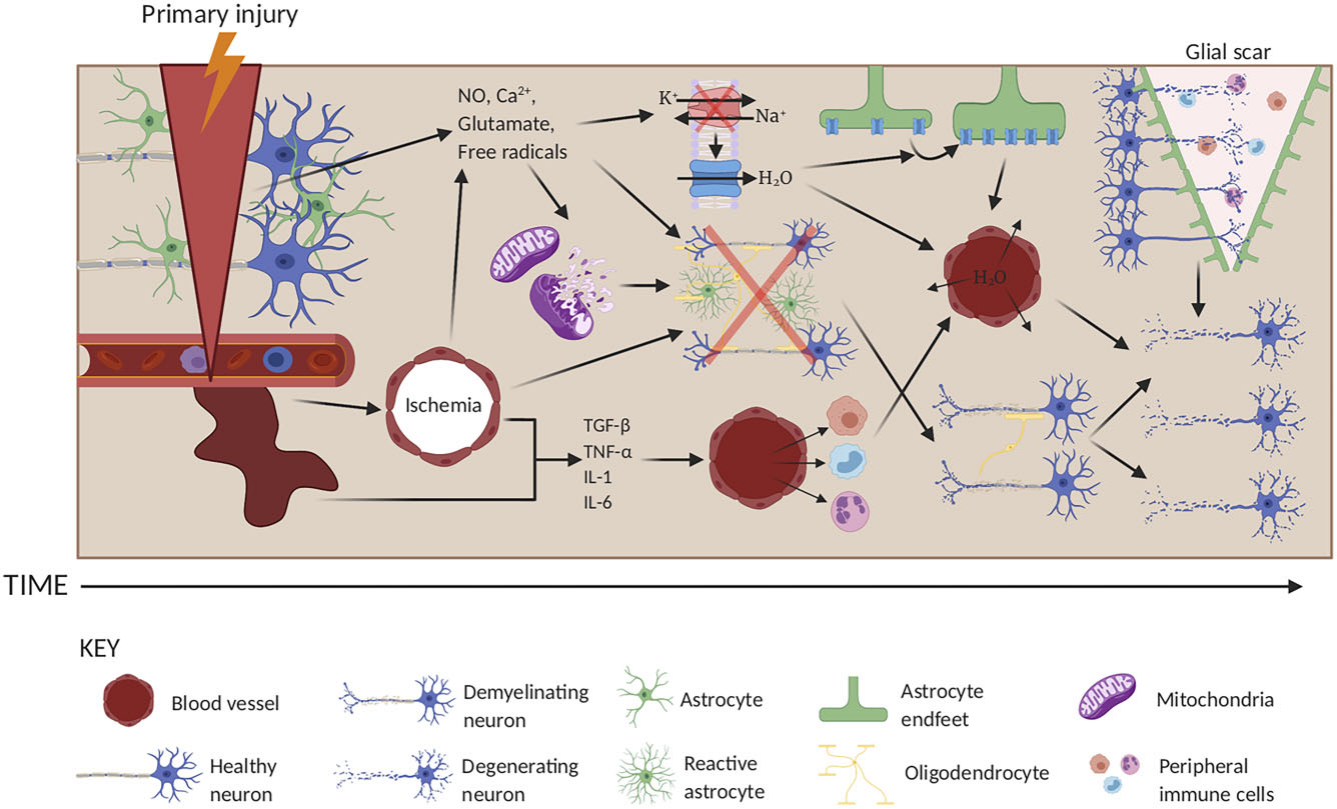
**The pathogenesis of traumatic injury in the CNS.** In the primary injury phase, the brain or spinal cord is injured following external insult. This primary injury results in mechanical damage to neurons, astrocytes, oligodendrocytes and blood vessels. A series of secondary injury cascades then occurs that potentiates the primary injury. In the earlier post-injury stages, damaged blood vessels may haemorrhage, resulting in ischaemia and release of inflammatory cytokines (e.g. TGF-β, TNF-α, IL-1, and IL-6). These cytokines attract blood-borne inflammatory cells such as neutrophils, macrophages and leucocytes, which act both to clear up cellular debris, but also cause further damage to healthy cells by enhancing local inflammation, eventually leading to neuronal loss from inflammatory damage and through Wallerian degeneration following oligodendrocyte death and demyelination. Damaged neurons may secrete free radicals, nitric oxide (NO), glutamate, and Ca^2+^, which further potentiate cellular damage by causing mitochondrial dysfunction leading to the loss of ATP, and by causing localized excitotoxicity. Collectively, these two events result in the loss of Na^+^/K^+^-ATPase activity and the loss of oxygen tension in astrocytes, which results in cytotoxic oedema through increased water absorption through AQP4 (blue). This is followed by ionic dysregulation, eventually leading to swelling via vasogenic oedema and cavity formation limited by the formation of a glial scar, which obstructs neuronal regrowth and enhances cell damage. Created using www.biorender.com.

Following a traumatic primary injury to the brain or spinal cord, a series of molecular cascades is triggered that results in further neuronal and glial cell death from inflammation, changes in brain energy metabolism and/or ischaemia/hypoxia, referred to as secondary damage.^73^^,^^74^ These molecular changes have architectural and functional consequences, including the development of oedema, the formation of glial scars and cavities, and neuronal cell loss.^75^ It is now clear that no single pathological feature can be explained in isolation in this complex process, which remains incompletely understood (Fig. 3). It is established that water flows into CNS tissue through AQP4, but the source of the water (whether the blood column, or the CSF/perivascular spaces) remains controversial.

**Figure 4:**
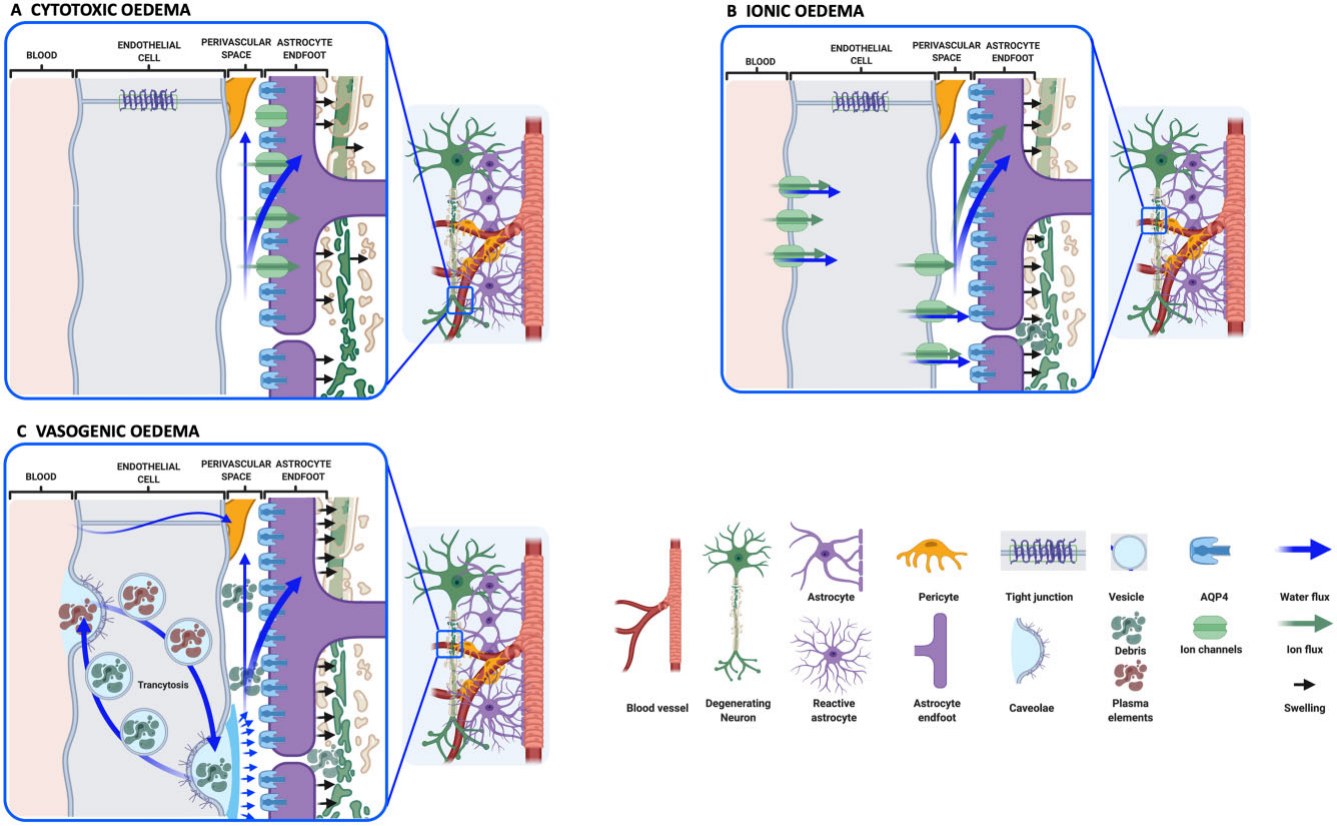
**Classification of CNS oedema.** (**A**) Cytotoxic oedema is defined by astrocyte swelling (black arrows) followed by neuronal dendrite swelling. The net entry of water (blue arrows), most likely from the perivascular space, is caused by disruption of cellular ion homeostasis (green arrows) following hypoxic insult. (**B**) Ionic oedema is characterized by transcapillary sodium ion and anion fluxes associated with cellular uptake of ions from the perivascular CSF, and entry of water into the brain parenchyma. Astrocytes continue to be swollen (black arrows) by water from the perivascular space and the vascular compartment. Neuronal death produces cellular debris in the extracellular space (ECS). (**C**) Vasogenic oedema is a result of BBB dysfunction, possibly following ionic oedema. Increased transcytosis may contribute to the entry of plasma elements (brown), followed by water. Clearance of debris from the ECS produced by neuronal cell death may also occur by transcytosis (green). In some severe cases, the tight junctions between the endothelial cells are weakened leading to increased permeability of cerebral blood vessels to plasma components. Created using www.biorender.com.

Oedema is a particular issue in the injured CNS because of the limited space (in the skull and spine) into which damaged tissue can swell. This is relevant following not only traumatic injury, but also in stroke and CNS tumours. In the last decade, the reclassification of oedema as cytotoxic, ionic or vasogenic (Fig. 4) based on observed changes in the brain has been widely adopted.^76^ Cytotoxic oedema (Fig. 4A) is defined as intracellular water accumulation without BBB disruption, usually as a consequence of the loss of oxygen tension. Morphologically, it is characterized by the swelling of astrocytes and the focal swelling of neuronal dendrites (known as beading).^77^^,^^78^ Ionic oedema (Fig. 4B) results from influx of water and sodium ions into the brain parenchyma prior to tight junction dysfunction, and is usually associated with cytotoxic oedema. Vasogenic oedema is a result of BBB dysfunction (Fig. 4C). The sources of water driving the formation of brain oedema remain a topic of debate.^58^^,^^76^^,^^79^^,^^80^ Methodological advances over the past decades, including two-photon microscopy and MRI, have led to new insights into the role of fluid in the perivascular spaces and the glymphatic system. In a recent study using a mouse ischaemic stroke model, the use of 22Na^+^ suggested that the CSF, not the blood, is the source of sodium ions.^58^ CSF was also identified as a major source of water driving AQP4-dependent oedema. Due to the incompressibility of CSF, enhanced influx of CSF into the parenchyma must be balanced either by enhanced secretion of CSF at the choroid plexus, enhanced drainage of CSF, or a change in the total volume of the ventricles and perivascular spaces. Temporarily limiting CSF secretion by targeting aquaporins or ion pumps in the choroid plexus membrane might therefore limit oedema formation in the short-term.

In traumatic brain injury and spinal cord injury, where the BBB/BSCB can be damaged directly by the primary injury (i.e. cytotoxic and vasogenic oedema co-exist), the source of water and sodium ions is likely to be a mixture of CSF and blood, with the exact ratio depending on the extent of BBB damage. Further endothelial dysfunction, secondary to the primary insult, leads to vasogenic oedema (Fig. 4C). For many years, it was proposed that BBB breakdown is required to facilitate the entry of plasma proteins into the extracellular space. However, more recent work has shown that vasogenic oedema can occur without physical rupture of endothelial cells.^81–83^ Although the suppression of transcellular transport (transcytosis) at the BBB is an active process that maintains a functional barrier, increased transcytosis observed in injured capillary endothelial cells may contribute to plasma protein entry, exacerbating brain swelling.^84^ Transcytosis may also be involved in the elimination of some proteins from the perivascular space back into the blood stream. Relocalization of AQP4 to the perivascular astrocyte membrane facilitates cytotoxic oedema,^7^ and may also increase the rate at which ionic oedema develops, both by increasing astrocyte membrane water permeability and possibly by regulating the endfoot membrane localization of ion channels via direct interaction (e.g. with Kir4.1, TRPV4, SUR1-TRPM4).^85–87^

Current available therapies for the treatment of brain oedema are hypertonic mannitol or saline, steroids for tumour-induced brain swelling and, once the oedema becomes life-threatening, decompressive craniotomy.^88^ The reliability and validity of the results of high-dose mannitol trials in the treatment of traumatic brain injury have been questioned^89^; a Cochrane review concluded that insufficient evidence was available to recommend mannitol for the management of traumatic brain injury patients.^90^ Although hypertonic saline is used to treat brain oedema following ischaemic stroke,^91^ a Cochrane review similarly reported that conclusions could not be drawn about the efficacy and safety of hypertonic saline or other intracranial pressure‐lowering agents in the management of acute traumatic brain injury.^92^ While the use of steroids did not reduce oedema following stroke,^93^ some success was reported in reducing brain tumour-associated oedema with dexamethasone.^94^ However, the molecular pathogenesis of tumour-associated oedema is quite different from that of trauma or stroke-associated oedema, as it is primarily driven by neoangiogenesis of vessels under-expressing tight junction proteins within the tumour.^95^ A recent study suggested that loss of AQP4 assembly into OAPs may facilitate evasion of apoptosis and enhanced migration in glioma cells,^96^ but how this interacts with tumour-associated oedema or AQP4 localization remains unexplored.

Little is known about mechanisms controlling the resolution of brain oedema. Early experiments showed that increased AQP4 expression was associated with oedema resolution,^77^^,^^97–101^ and in a vasogenic oedema model, *Aqp4*^−/−^ mice developed significantly increased intracranial pressure compared to wild-type mice, confirming a role for AQP4 in oedema resolution.^102^ Understanding this dynamic mechanism, including the role of the glymphatic system, will guide the development of new therapeutic approaches to treating oedema.

### Neuromyelitis optica

Neuromyelitis optica (NMO) is a rare but severe demyelinating autoimmune inflammatory condition of the CNS, formerly classified as a type of multiple sclerosis that primarily affects the optic nerve and spinal cord.^103^ The majority of NMO patients have autoantibodies against AQP4 (termed NMO-IgG) detectable in their serum.^104^ The mechanisms by which NMO-IgG cause the pathophysiological features of NMO remain elusive,^105^ although administration of NMO-IgG leads to NMO-like pathology in rodents,^106^^,^^107^ providing strong evidence that NMO-IgG is causative. However, different NMO-IgGs can have large differences in their ability to activate complement upon AQP4 binding, with some epitopes more facilitative for IgG hexamerization, meaning that there is not a simple relationship between antibody titre and disease severity.^108^ There is also evidence to support the idea that NMO-IgG facilitates both complement-dependent and complement-independent astrocytopathy.^109^^,^^110^ Most NMO-IgGs preferentially bind the M23 isoform of AQP4, but this selectivity appears to depend on an OAP assembly-associated conformation of the extracellular loops of AQP4, rather than a difference between the M1 and M23 proteins *per se*.^111^^,^^112^ The effect of NMO-IgG on OAP size is unclear; one study reported an increase in average OAP size following NMO-IgG binding,^113^ another found no effect^114^ and a third reported a decrease in average OAP size.^19^ More recent work suggests that changes in the dynamics (rather than the average size of OAPs) may be altered by NMO-IgG, with potential consequences for glutamatergic synapse function.^19^ Similarly, whether NMO-IgG inhibits AQP4 water channel function is controversial^113^^,^^114^; an exquisitely tight seal between the extracellular domain of AQP4 and NMO-IgG would be required to inhibit water transport. The potential effects of NMO-IgG on AQP4-mediated glymphatic function remain unexplored.

### Neuroinflammatory disorders

AQP4 may also have a role in CNS inflammation in a manner that is independent of autoantibody formation. AQP4 expression, either on peripheral immune cells, or on CNS astrocytes may regulate CNS immune cell migration and trafficking, or glial activation and cytokine production, respectively.^115^ One study using *Aqp4*^−/−^ mice reported that the central neuroinflammatory response to CNS lipopolysaccharide (LPS) injection, including TNFα release, was reduced, suggesting a pro-inflammatory role for AQP4.^116^ In a more recent study, *Aqp4* gene deletion altered astroglial cytokine release and exacerbated α-synuclein pathology in a rodent model of Parkinson’s disease.^117^ These studies suggest that AQP4 may function to regulate CNS cytokine signalling. Given the role of AQP4 in glymphatic clearance,^37^^,^^56^ one possible explanation for these findings is that AQP4-dependent glymphatic exchange contributes to the distribution and clearance of cytokines within the CNS. The impacts that physiological and pathological changes in AQP4 localization have on its inflammatory roles remain to be defined.

### New horizons for drug discovery

IMD-0354/AER-270, TGN-020, acetazolamide, budesonide, furosemide, and various anti-epileptics have all been proposed to be AQP4 inhibitors on the basis of data primarily derived from the *Xenopus laevis* oocyte swelling assay.^40^ When retested in transport assays using primary astrocytes expressing endogenous AQP4, mammalian cell lines overexpressing exogenous AQP4 or recombinant AQP4 protein, many putative pore-blockers have been found to lack AQP4 inhibitory function.^40^^,^^118^^,^^119^ It therefore remains unclear, after several decades of effort, whether a specific AQP4 pore-blocking inhibitor can be developed, providing impetus to explore alternative strategies, such as targeting dynamic AQP4 subcellular relocalization.

Several lines of evidence over the last decade have also highlighted the diverse functions of aquaporins beyond water homeostasis.^10^ AQP4 has been proposed to associate with various ion channels in the astrocyte membrane, including the inwardly rectifying potassium channel Kir4.1,^120^ the mechanosensitive cation channel TRPV4,^121^ and the ABC protein/TRP channel complex SUR1-TRPM4.^122^ AQP4 and Kir4.1 are co-localized in astrocyte membranes,^123^ and co-immunoprecipitate from glial cells.^120^ This interaction was proposed to support potassium ion spatial buffering by astrocytes after neuronal activity,^124^ and cellular potassium ion reuptake is delayed in *Aqp4*^−/−^ mice in an epilepsy model,^125^ although it still unclear whether there is a functional relationship between AQP4 and Kir4.1.^126^ However, there is some evidence that Kir4.1 limits the osmotic swelling of spinal cord astrocyte processes.^127^^,^^128^ In several cell types, TRP channel plasma membrane trafficking is dependent on the expression of an aquaporin protein.^10^ AQP4 and the SUR1-TRPM4 monovalent cation channel complex co-immunoprecipitated when overexpressed in COS-7 cells, preactivation of SUR1 with diazoxide increased astrocyte swelling in response to a calcium ionophore, and SUR1-TRPM4 was upregulated and TRPM4 knockout blocked astrocyte swelling in a mouse cerebellar cold injury model.^122^ Furthermore, inhibition of the SUR1-TRMP4 complex using glyburide reduces oedema formation in multiple rodent models of brain pathology.^74^ This work raises the intriguing possibility that as well as directly regulating astrocyte membrane water permeability, AQP4 facilitates membrane insertion of oedema-associated TRP channels. Based on the new molecular understanding of the role and mechanisms of dynamic AQP4 subcellular relocalization and protein–protein interactions in CNS oedema, novel anti-oedema therapies are likely to emerge. This is of the utmost importance because there are currently no pharmacological tools to prevent or reduce CNS oedema. Treatment therefore focuses on symptom management, which is only possible after the oedema has developed (and has caused secondary damage) and which uses interventions developed decades ago. These new possibilities for drug discovery offer new hope to the millions of people annually affected by CNS oedema and neurodegenerative diseases.^7^

The dependence of the field on static, *in vitro* models rather than dynamic, *in vivo* visualization may have contributed to both the glymphatic system and AQP4 subcellular relocalization remaining undiscovered for so long. Recapitulating the complex structure and function of the BBB *in vitro* is challenging. Rodent *in vivo* studies and slice cultures are anatomically more realistic, but are hampered by species differences in BBB function and by the isolation of tissue slices from the blood circulation, peripheral immune actors, and both CSF and intracranial pressure dynamics. New developments in 3D tissue engineering, organ-on-a-chip technologies, and induced pluripotent stem cell differentiation may help the field to begin to address some of these limitations.^129–131^ Future gains in our understanding of astroglial and AQP4 contributions to CNS physiology, and how their dysfunction contributes to the development of CNS disease, will likely depend on the combined use of emerging *in vitro* techniques, such as BBB/glymphatics-on-a-chip, to dissect specific physiological processes along with dynamic *in vivo* approaches that preserve the full anatomy and physiology of the glial–vascular unit.

## Funding

We acknowledge grants from the Biotechnology and Biosciences Research Council (to R.M.B., A.C.C., and P.K. through BB/P025927/1), Aston University (to P.K. through a 50^th^ Anniversary Prize Fellowship), the Swedish Research Council (to S.T.-H. through 2013–05945); the Crafoord Foundation (to S.T.-H. through 20140811 and 20180916) and the Magnus Bergvall Foundation (to S.T.-H. through 2015–01534).

## Competing interests

The authors report no competing interests.

## Abbreviations

BBB: blood–brain barrier
BSCB: blood–spinal cord barrier
NMO: neuromyelitis optica
OAPs: orthogonal arrays of particles
